# Treatment with infliximab and tacrolimus in steroid-refractory pneumonitis secondary to anti-HER2 therapy

**DOI:** 10.1016/j.esmoop.2024.104128

**Published:** 2025-01-22

**Authors:** O. Fakih, E. Ahmed, M. Paravasthu, J. Pearson, L.G. Spencer, C. Palmieri

**Affiliations:** 1 The Clatterbridge Cancer Centre NHS Foundation Trust, Liverpool; 2 Liverpool Interstitial Lung Disease Service, Liverpool University Hospitals NHS Foundation Trust, Aintree Site, Liverpool; 3 Department of Molecular and Clinical Cancer Medicine, Institute of Systems, Molecular and Integrative Biology, University of Liverpool, Liverpool, UK

**Figure 1:**
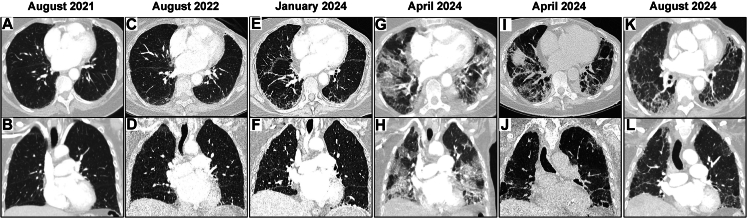
**CT scans of thorax before and during treatment with anti-HER2 therapies and subsequent presentation with pneumonitis.** (A and B) Minimal sub-pleural changes related to prior radiotherapy before any anti-HER2 therapy. (C and D) Mild, non-specific sub-pleural ILAs before commencing T-DXd. (E and F) Mildly progressive ILST in the mid to lower lungs before discontinuing T-DXd. (G and H) Bilateral extensive GGOs and scattered areas of air-space consolidation in keeping with pneumonitis. (I and J) Interval partial improvement in the extent of GGOs and consolidation with residual scattered peribronchovascular reticular opacities and resolving consolidation following treatment with CSs, infliximab and tacrolimus. (K and L) Further improvement of inflammatory lung changes with residual ILST and fibrosis, and traction bronchiectasis. CS, corticosteroid; CT, computed tomography; GGO, ground-glass opacity; HER2, human epidermal growth factor receptor 2; ILA, interstitial lung abnormality; ILST, interlobular septal thickening; T-DXd, trastuzumab deruxtecan.

We report the case of a 76-year-old woman with estrogen receptor-positive, human epidermal growth factor receptor 2 (HER2)-positive metastatic breast cancer (BC), involving the liver and bones, who had previously been treated with paclitaxel in combination with trastuzumab and pertuzumab (TP) (October 2021-July 2022), trastuzumab emtansine (T-DM1) (September 2022-November 2022) and subsequently trastuzumab deruxtecan (T-DXd) (December 2022-February 2024); cross-sectional imaging of the thorax was carried out during this period (Figure 1A-F). She was an ex-cigarette smoker, having stopped at age 35 years, and had normal renal function. In January 2024, computed tomography cross-sectional imaging suggested progressive liver disease, which was confirmed by magnetic resonance imaging of the liver in February 2024, and she was subsequently commenced on trastuzumab and tucatinib, capecitabine being omitted pending dihydropyrimidine dehydrogenase genotyping. Four weeks after commencing treatment, she presented with a 6-day history of fatigue, progressive dyspnoea and dry cough. On admission, she was tachypnoeic and apyrexial and had an oxygen saturation of 88% on room air. Cross-sectional imaging demonstrated extensive ground-glass opacities (GGOs) and scattered areas of air-space consolidation (Figure 1G and H). She was commenced on intravenous (i.v.) methylprednisolone (MP) 1 mg/kg as well as piperacillin/tazobactam. At day 3 following presentation despite steroid and antimicrobial treatment, her oxygen requirements increased to 6 l/min; given this, MP was increased to 1 g i.v., mycophenolate mofetil (MMF) 500 mg was commenced twice daily and antibiotic cover broadened with clindamycin, primaquine and doxycycline. A screen for alternative causes of inflammatory lung pathology, including respiratory and blood-borne viruses, atypical bacteria, fungal organisms and auto-antibodies, was negative. Bronchoscopy was not carried out due to high oxygen requirements. On day 5, the patient had progressive respiratory failure with increased breathlessness and an oxygen requirement of 50% oxygen at 45 l/min, MMF was substituted for tacrolimus at 3 mg twice daily and a single dose of infliximab 5 mg/kg was administered. By day 7, her oxygen requirements decreased, with a slow improvement of her dyspnoea. Due to a lack of evidence of infection, antimicrobial therapies were then discontinued. At day 10, i.v. MP was weaned to oral prednisolone 100 mg with further tapering of dose until completion at day 56. Repeat cross-sectional imaging at day 14 post-admission showed improvement in the extent of GGOs and consolidation, with residual scattered peribronchovascular reticular opacities (Figure 1I and J). A further dose of infliximab 5 mg/kg was given 14 days after the initial dose, and the patient was discharged home on day 36 with an ambulatory oxygen requirement of 2 l/min (28% oxygen). Her tacrolimus was continued until cessation of prednisolone on day 56. A review of imaging demonstrated no evidence of any interstitial lung changes during treatment with TP and T-DM1. However, progressive interstitial lung changes were noted over a period of 15 months during treatment with T-DXd up until its discontinuation, albeit with no reported symptoms (Figure 1E and F). Following discharge, there was progressive improvement with a reduction in the need for ambulatory oxygen. Most recent cross-sectional imaging demonstrated ongoing improvement in the bilateral inflammatory lung changes, with residual fibrosis (Figure 1K and L). Follow-up imaging in August 2024 demonstrated extensive progression of the liver metastases, with further improvements in the thorax. Given this, in September 2024, after discussion with the patient, trastuzumab and tucatinib was recommenced with capecitabine. Three cycles of treatment have been given and follow-up imaging is awaited. At a clinic visit in November 2024, the patient had continued symptomatic improvement with normal saturations at rest, and a requirement for ambulatory oxygen only on strenuous exertion.

Anti-HER2 therapy is associated with interstitial lung disease (ILD)/pneumonitis and is recognised with trastuzumab alone; the prescribing information for trastuzumab contains a black box warning and documents a rate of 0.7% with fatal cases reported.^1^ A pooled analysis of BC patients within T-DXd studies reported an ILD/pneumonitis rate of 15% with deaths in ∼3%.^2^ The mechanism underlying ILD with T-DXd is unclear, the only pathophysiology data coming from one animal study.^3^ This study reported no lung toxicity with deruxtecan alone, in contrast with T-DXd administration, which resulted in uptake by non-HER2-expressing alveolar macrophages with none localising in the HER2-expressing pulmonary epithelial cells. In the current case, the acute presentation was preceded by a short period of treatment with trastuzumab and tucatinib; the role of these in exacerbating the radiologically evident but subclinical ILD is unclear. Given the association of trastuzumab with ILD and the use of trastuzumab with tucatinib and capecitabine post-T-DXd in the metastatic setting, research into the possible risks of ILD with trastuzumab following prior T-DXd treatment is needed, particularly where T-DXd is discontinued due to ILD. This is particularly important as the current safety data for trastuzumab, tucatinib and capecitabine are based on prior T-DM1 treatment. To date, as far as we are aware, there are no data in regard to the risk of ILD following T-DXd with trastuzumab, tucatinib and capecitabine or any other anti-HER2 therapy. In the current case, given the clinical need and after discussion, trastuzumab and tucatinib was recommenced with capecitabine, to date with no respiratory issues.

Established guidelines for immune-checkpoint inhibition (ICI)-induced ILD that is resistant to steroid therapy suggest the use of additional immunosuppressive therapies such as MMF, tacrolimus or disease-modifying biologics such as tumour necrosis factor (TNF) inhibition with infliximab.^4^ These recommendations formed the basis for the introduction of additional immunosuppressant in the current case, given her clinical deterioration and lack of response to steroids. Early treatment with infliximab in ICI-ILD has been reported to be clinically beneficial.^5^ In regard to T-DXd, there is no reference to the management of steroid-refractory ILD in the summary of product characteristics or prescribing information, and currently, there are no recommendations relating to this scenario namely ILD secondary to anti-HER2 therapy. Given the reported activity of TNF inhibition strategies in ICI-ILD and the risk of death associated with T-DXd ILD, consideration should be given to using these in line with the published European Society for Medical Oncology (ESMO) guidance for ICI-ILD in steroid-refractory T-DXd ILD in the absence of any other active therapeutic strategy and to the development of studies formally looking at their efficacy. In summary, we present a case of ILD secondary to anti-HER2 therapy treated with infliximab and tacrolimus, with good clinical and radiological response. This case highlights the need for research and guidance in relation to alternative strategies in steroid-refractory ILD, the need to be vigilant to ILD on as well as following T-DXd cessation and the need for safety data in regard to the use of combination anti-HER2 therapy following T-DXd.
